# Sexual differences in defensive strategies: investigating chemical defences and visual signals in a wasp moth *Amata nigriceps*

**DOI:** 10.1098/rsos.242186

**Published:** 2025-04-23

**Authors:** Georgina E. Binns, Liisa Hämäläinen, Hannah M. Rowland, Lorenzo Caputi, Maritta Kunert, Johanna Mappes, Giovanni M. Ramon-Cabrera, Kate D. L. Umbers, Nathan S. Hart, Marie E. Herberstein

**Affiliations:** ^1^ School of Natural Sciences, Macquarie University, Sydney, New South Wales, Australia; ^2^ Department of Biological and Environmental Sciences, University of Jyväskylä, Jyvaskyla, Finland; ^3^ Max Planck Institute for Chemical Ecology, Jena, Germany; ^4^ Department of Evolution, Ecology and Behaviour, University of Liverpool, Liverpool, UK; ^5^ Department of Natural Product Biosynthesis, Max Planck Institute for Chemical Ecology, Jena, Germany; ^6^ University of Helsinki, Helsinki, Finland; ^7^ Universidad San Francisco de Quito USFQ, Colegio de Ciencias Biológicas y Ambientales, Instituto de Biodiversidad Tropical iBIOTROP, Museo de Zoología & Laboratorio de Zoología Terrestre, Quito, Ecuador; ^8^ Hawkesbury Institute for the Environment, Western Sydney University, Sydney, New South Wales, Australia; ^9^ School of Science and Health, Western Sydney University, Sydney, New South Wales, Australia; ^10^ Centre for Taxonomy and Morphology, Leibniz Institute for the Analysis of Biodiversity Change, Bonn, Nordrhein-Westfalen, Germany; ^11^ Department of Biology, University of Hamburg, Hamburg, Germany

**Keywords:** aposematism, Australia, tiger moths, plant alkaloids, metabolomics, predator defences

## Abstract

Aposematic animals use conspicuous warning signals to advertise their chemical defences to predators. Selection by predators can favour conspicuousness and large pattern elements, which enhance predator avoidance learning. In aposematic species, conspicuousness often varies among individuals. This variation can be explained if conspicuousness reflects the levels of chemical defences, if signal production or defence acquisition is costly, and if physiological trade-offs and opposing selection pressures impose constraints. To understand the link between conspicuousness and chemical defences, we need to quantify the variability in warning signals and identify the chemical compounds involved. Here, we examined the warning signal variability and chemical composition of the red-necked wasp moth (*Amata nigriceps*). We photographed the wings and abdomens of male and female moths and analysed their chemical composition using ultra-performance liquid chromatography. Females displayed more orange on their wings, a trait known to enhance protection against predators. While we ruled out the presence of pyrrolizidine alkaloids in adult moths, an untargeted metabolomics approach suggests that they sequester other compounds, such as steroidal alkaloids and alkylbenzenes, which may serve as chemical defences. Females had higher concentrations of these compounds than males but ecotoxicology assays with *Daphnia* showed that male and female moths exhibited similar levels of toxicity.

## 1. Introduction

Aposematism is an antipredator strategy in which a species advertises its unprofitability to predators through a conspicuous warning signal [1,2]. Selection by predators favours large and conspicuous colours and patterns because they can elicit innate wariness in predators [3] and are more readily learned and remembered by predators [4,5]. Variation in warning signals is, however, widespread [6], and recent work has shown how additional selective pressures including environmental and physiological constraints, as well as the effect of sexual selection can affect warning signal variation [7–10].

To study the ecological and evolutionary trade-offs between signal expression and fitness, it is important to link them with physiological and ecological mechanisms that affect signal expression [11]. If acquiring secondary defences is costly [12], then trade-offs between warnings signals, chemical defences and other life-history traits might occur [11,13–17]. To understand possible trade-offs, it is important to know the biochemical composition of aposematic traits [18]. Here, we aimed to characterize the variation in warning signals of the red-necked wasp moth (*Amata nigriceps*) and the composition of secondary defences, to provide insights into the possible physiological mechanisms underlying warning signal variation.

**Figure 1.**
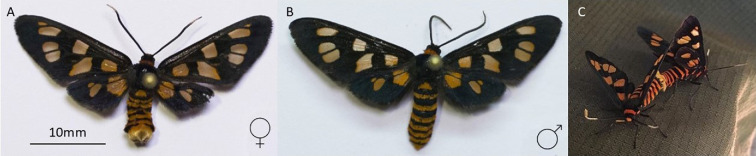
*Amata nigriceps* moths: (A) female, (B) male and (C) a mating couple, female below and male above. Both (A) and (B) moths were collected from the same population in Macquarie Park, Sydney, New South Wales. Mating couple observed in Ku-ring-gai Reserve, Sydney. Images: G.E.B.

The red-necked wasp moth *A. nigriceps* (Lepidoptera: Erebidae: Arctiinae) is a diurnal species distributed along the east coast of Australia. *A. nigriceps* has striking orange wing spots and abdominal stripes, contrasted against a black background, which gives it the appearance of mimicking a wasp (figure 1). It has a conspicuous and nonchalant (*sensu* [19]) flight pattern and has been suggested to use pyrazines as a chemical defence [20]. We have previously shown that the size of the orange wing spots varies within and between populations from 10 to 30% of the total wing area and that females have larger proportions of orange in the wings than males [21]. In addition to spot size, the number of orange abdomen bands varies, with males having one more band than females (male bands = 6, female bands = 5; figure 1). *Amata nigriceps* moths with smaller wing spots face a higher risk of predation compared to those with larger wing spots, particularly when their orange abdominal stripes are not visible, and moths with fewer stripes on the abdomen are more susceptible to predation than those with a greater number of stripes [22]. Whether these differences in warning signals and predation risk are associated with different levels of toxicity has not been tested.

Sex differences in warning signals have been reported in many species (e.g. damselflies [23]; poison frogs [24]; burnet moths [25]; and tiger moths [26]). These differences can be explained if one sex is more active or behaviourally conspicuous and therefore has increased encounter rates with predators, which can strengthen selection for a more conspicuous warning signal [27,28]. There is also growing evidence for the interplay between natural and sexual selection on warning signal variation [23,28]. For example, in the aposematic wood tiger moth *Arctia plantaginis*, yellow males are typically better protected from avian predators, whereas white males are more successful in mate attraction, and this trade-off can explain the coexistence of the two different colour morphs [28]. Sexual selection might also contribute to differences in warning signal expression in males and females. For example, strawberry poison frog (*Oophaga pumilio*) males are brighter than females, which can be explained by female preference for bright males [8]. While male sexual signalling and competition may explain why some males are more conspicuous than females, it does not explain why female *A. nigriceps* display larger warning signals than males (e.g. [21]).

Sexes can also differ in the composition and/or quantity of the chemical defence [29]. Defence chemicals can be synthesized de novo, such as the biosynthesized alkaloids found in some species of *Pseudophryne* poison frogs [30]. Alternatively, they can be sequestered from food plants, such as in the arctiine moths of the Northern Hemisphere that sequester pyrrolizidine alkaloids (PA) as part of their chemical defences (e.g. [31–33]). In six-spot burnet moths (*Zygaena filipendulae*), larger females possess a higher quantity of cyanogenic glucosides, but when controlling for weight, the concentration of toxins does not differ between the sexes [25]. In contrast, in cotton harlequin bugs (*Tectocoris diophthalmus*), the total amount of toxin is similar in males and females which, when controlling for weight, leads to a higher toxin concentration in males [34]. The composition and variability in the chemical defences of *A. nigriceps* have not until now been studied.

Here, we were interested in quantifying the sex-specific warning signals and chemical defences of the red-necked wasp moth *A. nigriceps*. Given the potential connections between chemical defences and variable warning signals [35], we first quantified the morphological differences between male and female *A. nigriceps* by measuring body mass, wing length and wing and abdomen colour patterns, and then we sought to identify the chemical defences using ultra-performance liquid chromatography (UPLC). Because PA have previously being detected in arctiine moths, we used a targeted approach to determine whether this species utilizes these defensive compounds. More specific information regarding the biochemical composition of the moth’s chemical defence could help determine whether the moths biosynthesize or sequester defences or if they use a mixture of both strategies. We also conducted ecotoxicology assays to determine the toxicity of these moths. We hypothesized that females might be under stronger selection to deploy chemical defences and their associated warning signals because carrying eggs not only makes them less agile flyers but also makes them a more nutritious meal for predators compared to males. We therefore predicted that females would be more toxic than males and that they would have more diverse chemical compounds. Our aim is to provide insight into the possible ecological and physiological trade-offs associated with the production of aposematic traits.

## 2. Methods

### 2.1. Study species

The red-necked wasp moth *A. nigriceps* is diurnal, polyphagous and displays characteristic orange ‘tiger’ moth spots and stripe markings that contrast conspicuously with a black background. Little is known about the species’ ecology, although it has been suggested that larvae feed on decaying leaf litter [36]. We have reared larvae successfully in the laboratory on dandelion leaves [21]. The moths are bivoltine and emerge as adults to mate during two seasons: October–December and February–April. The distribution of this species ranges along the east coast of Australia from Brisbane, Queensland, through New South Wales (NSW) and Victoria, and the moths can be found in coastal and urban-disturbed areas, temperate bushland and rainforest [21].

#### 2.1.1. Sample collection

Live *A. nigriceps* adults were collected using butterfly nets from Wallumattagal land at Macquarie Park, NSW (33°46′25″ S, 151°06′45″ E; *n* = 139, females = 31, males = 108) and Guringai land at Wyoming, NSW (33°24′23″ S, 151°21′37″ E; *n* = 69, females = 18, males = 51) between March and November 2020. Individual moths were transported alive in separate containers to Macquarie University, where they were euthanized by placing them in a freezer at −30°C.

#### 2.1.2. Image analysis

We measured forewing length, the proportion of orange in the wing and the proportion of orange in the abdomen. All collected moths (*n* = 207) were included in the wing length and colour analysis. Individuals were briefly removed from freezer storage to remove one set of forewings and hindwings and then returned to the freezer. Twenty females (Macquarie Park: *n* = 17, Wyoming: *n* = 3) and 20 males (Macquarie Park: *n* = 10, Wyoming: *n* = 10) were used to measure the abdominal stripes, with the remaining abdomens returned to the freezer for chemical analyses and ecotoxicology assays.

Wings were placed on matte white paper and photographed with a Canon EOS 5D single-lens reflex camera with a 100 mm Canon MP-E lens illuminated with a BK Plus System (Bun, Inc.). Images were white balance corrected and then cropped in Adobe Photoshop (v 19.1.5). For each moth, either the left or right set of wings was selected for analysis based on preservation quality (left wings: *n* = 117, right wings: *n* = 90). We measured forewing length by calculating the distance between the shoulder and the wing tip using ImageJ measurement function (v 1.52a [37]). We measured the proportion of the orange in the wings in relation to the black wing area using the colour adjacency package in pavo [38] in Rstudio (v 3.4.2 [39]). This gave a value we term ‘*pSpot*’—with higher *pSpot* values indicating a higher proportion of orange. All images were inspected for accuracy of colour mapping by GEB, and for those images in which pavo had incorrectly assigned colour, e.g. black where the original image is orange (*n* = 135), the image pixels were colour corrected using the ‘Clone Stamp’ tool in Photoshop (see electronic supplementary material for example of output) and then re-analysed in pavo (see [21] for method details).

Whole moths for abdominal measurements were thawed at 5°C for 24 hours and then pinned through the thorax with a size 3 insect pin. Legs and antennae were cut off using microdissection scissors and discarded. The specimens were then laid flat with their abdomen in contact over an extruded polystyrene foam board that was wrapped in white paper. To ensure that the abdomen was flat and most of the surface of the dorsal tergites was visible, we used feather-light forceps to pull the abdomen by the pleura from the last two tergites. We then secured the abdomen in that position by inserting a micropin (0.193 × 15 mm) across the second to last (black) tergite and into the board. We left the specimens to air-dry at room temperature for at least 3 days before removing the micropin. In some cases, the abdomen was damaged during pinning, or we did not manage to pin it straight, and we had to discard these samples (*n* = 7), resulting in a final sample size of 16 females and 17 males. To take the photographs, we placed the pin-mounted specimens on a small white foam board under a Leica M205 FA stereo microscope (Leica Microsystems) with a maximum magnification of 159× illuminated by a dual gooseneck fibre optic light (Leica LED5000 SLI, Leica Microsystems). We took z-stacked photographs of each specimen’s abdomen using the Z-Stack module in the Leica Application Suite X version 3.3.3.16958 (Leica Microsystems). Each image was created by stacking 20 photographs taken at equidistant steps by the motorized stereomicroscope from the abdomen’s lowest focal plane up to the highest focal plane. We included a 1-mm scale bar in each photograph. We standardized the colour temperature of each photograph, using the ‘Color Sampler’ tool in Adobe Photoshop (v 24.1.1) and the ‘Channel Mixer’ tool was used to match the values at each channel to the white colour values on the RGB scale (R = 255, G = 255, B = 255).

Because the tip of the abdomen was often slightly twisted or damaged, we used only the middle part of the abdomen for the colour analysis, including four orange and four black stripes for females and five orange and five black stripes for males (see electronic supplementary material, figure S1). We calculated the proportion of orange in the abdomen (hereon described as ‘*pStripe*’) using the same colour adjacency package in pavo as was used in the wing colour analysis.

### 2.2. Extraction of *A. nigriceps* for targeted and untargeted metabolomics analysis

Thirteen male and 13 female *A. nigriceps* moths (all from Macquarie Park) were individually freeze-dried in 1.5 ml Eppendorf tubes and stored at room temperature prior to analysis. The samples were shipped to the Max Planck Institute for Chemical Ecology where they were pulverized using a TissueLyser II (Qiagen) and tungsten carbide beads (3 mm) at room temperature and extracted using 1 ml of LC/MS grade methanol (CAS number 67-56-1) containing 2 mM ajmaline (Extrasynthese, CAS number 4360-12-7) as internal standard. The samples were sonicated for 10 minutes and then vortexed for 20 minutes followed by centrifugation to remove the debris. The supernatant was removed and filtered through 0.2 µM PTFE filters. Ten microlitres of each sample were combined into the quality control sample (QC). Seventy microlitres of each sample was used for untargeted metabolomics analysis while the remaining extract was dried under vacuum. The dried samples were reconstituted in 1 ml of 0.05 M H_2_SO_4_ and processed according to the PA extraction protocol described by Betteridge *et al*. [40] with the following modifications. Briefly, the samples were sonicated for 15 minutes at 40°C, centrifuged at 4000*g* for 10 minutes and then applied onto Strata SCX (Phenomenex) 100 mg/1 ml cartridges previously conditioned with 2 ml of 0.05 M H_2_SO_4_. After washing the cartridges with 2 ml of water followed by 2 ml of methanol, the compounds were eluted with 2 ml of ammonia/methanol (94 : 6 v/v). The samples were dried under nitrogen and then reconstituted in 100 µl of methanol prior to targeted metabolomics analysis for PA detection.

### 2.3. Untargeted metabolomics identification

We performed a UPLC/MS analysis on an Ultimate 3000 (Thermo Scientific) UPLC system coupled to an Impact II (Bruker) qTOF mass spectrometer. A Phenomenex Kinetex XB-C18 column (100 × 2.1 mm, 2.6 µm; 100 Å) kept at 40°C was used for the chromatographic separation. At the beginning of each run, the first 0.3 minutes of liquid chromatography input was redirected to waste. The solvent system was A = 0.1% HCOOH in water and B = ACN. The column was conditioned with 1% solvent B before injection of the samples. From 0 to 0.5 minutes, B was kept at 1%. Between time 0.5 and time 7 minutes a linear gradient from 1 to 100% was run, after which the column was kept at 100% B for 2 minutes to elute all compounds. The column was then re-equilibrated at 1% B for 3 minutes before the next injection. The total chromatographic time was 12 minutes. The flow rate was 0.6 ml min^−1^ and 2 µl of samples was injected for the analysis. The mass spectrometer was operated in positive ionization mode using an electrospray source. Source parameters were as follows: cone voltage 3.5 kV, nebulizer 2.5 bar, dry gas 11 l min^−1^ and dry temperature 250°C. The acquisition range was set from 120 to 1000 *m*/*z*. Each run started by injecting a sodium formate–isopropanol solution at 0.2 ml h^−1^ for the first 60 s. Subsequently, we recalibrated the *m*/*z* values based on the expected cluster ion *m*/*z* values, redirecting the LC input to waste. A UPLC/MS chromatogram showing metabolite profiles can be found in the electronic supplementary material, figure S2.

### 2.4. Targeted ultra-performance liquid chromatography/mass spectrometry analysis of pyrrolizidine alkaloids

Targeted analysis of PA was performed using an Ultimate 3000 UPLC system coupled to a Bruker EVOq triple quadrupole mass spectrometer. Chromatographic separation was performed on a Phenomenex Kinetex XB-C18 column (100 × 2.1 mm, 2.6 µm; 100 Å) kept at 40°C. The solvent system was A = 0.1% HCOOH in water and B = ACN. The column was conditioned with 5% solvent B before injection of the samples. A linear gradient from 5 B to 100% B in 6 minutes was used for the separation of the compounds, after which the column was washed and re-equilibrated at 5% B for 3 minutes before the next injection. The total chromatographic time was 12 minutes. The flow rate was 0.6 ml min^−1^ and 2 µl of samples was injected for the analysis. The mass spectrometer was operated in positive ionization mode using an electrospray source. Source parameters were as follows: source temperature 450°C, cone voltage 3.5 kV, cone gas flow 20 arbitrary units, nebulizer gas flow 50 arbitrary units and cone temperature 350°C. Unit resolution was applied to each quadrupole. We optimized the multiple reaction monitoring (MRM) by flow injecting the standards (see electronic supplementary material, table S1). We experimentally determined the cone voltage, adjusting the collision energies automatically (MS Workstation software 8.2; Bruker). Each MRM transition was set with a dwell time of at least 25 ms. The standard compounds (electronic supplementary material, table S1) used in this study were: seneciphylline N-oxide (PhytoLab), monocrotalin N-oxide, senecionine N-oxide, senkirkine, senecivermine, heliotrine, adynerine and Lycopsamine (all supplied by ChemFaces).

### 2.5. Ecotoxicology assays

We used common water fleas (*Daphnia carinata*), obtained from aquaticlivefood.com.au as a test organism to investigate toxicity differences between male and female *A. nigriceps. Daphnia* are a frequently used organism for ecotoxicology studies due to their high sensitivity to various chemicals and have been recommended by various authors for standardizable test assays (e.g. [41,42]). Although *Daphnia* are not natural predators of the moths, they are highly sensitive to chemicals and commonly used in ecotoxicology assays, including toxicity studies with aposematic insects [34,43]. We extracted 15 female and 15 male *A. nigriceps* (all from Wyoming), following the protocol described in Arenas *et al*. [43] and Medina *et al*. [34]. The wings of the frozen moths were removed, and the moths were weighed to the nearest 0.0001 g using a digital scale (Mettler Toledo GmbH). We macerated the body tissue with a plastic pestle in a 1.5 ml Eppendorf tube and added 1 ml 100% methanol (Sigma Aldrich). The tube was spun in a centrifuge at 14 500 r.p.m. for 10 minutes, the supernatant was extracted into a new 1.5 ml Eppendorf tube, and the pellet was discarded. The excess methanol was evaporated using a vacuum concentrator (miVac Quattro concentrator, Genevac Ltd) set at 45°C for 2.5 hours. Once there was no visible methanol left in the sample, we created a stock extract by adding 1.5 ml MilliQ water and homogenizing the sample by vortexing it for 20 s.

Our pilot assays suggested that a 20% concentration was suitable for assessing toxicity differences (see electronic supplementary material, figure S3), so we prepared 0.5 ml test solutions of each moth (*n* = 30) by adding 0.1 ml stock extract and 0.4 ml MilliQ water to a 2 ml Eppendorf tube. Following Arenas *et al*. [43], we also prepared control solutions that included (i) 0.5 ml MilliQ water (*n* = 6) and (ii) 0.5 ml 5% methanol solution (*n* = 7). These were included to control mortality caused by handling *Daphnia* and potential toxic effects of residual methanol that might have been left in the samples after evaporation. All solutions were vortexed and kept in a fridge (−4°C) overnight. The assays were conducted on the following day by placing 10 adult *Daphnia* in the solutions and recording the number of dead individuals at 10 minutes, 30 minutes, 1 hour and then every hour until the 4 hour mark. To ensure that the toxicity assays were reproducible, we tested most of the moths twice (12 females and 14 males) and calculated the repeatability of the two assays.

### 2.6. Statistical analyses

#### 2.6.1. Sex differences in morphology and warning signals

Sex differences in forewing length (mm) and in the proportion of orange in the wings (*pSpot*) and abdomen (*pStripe*) were analysed using generalized linear models. Explanatory variables in each model included sex and the locality where the moths were collected (Macquarie Park/Wyoming). To investigate whether sex differences were similar in both localities, the first models for wing measures also included an interaction term between sex and population. Because of a lower sample size of abdomen images and an unequal representation of females and males in each location, this interaction term was not included when analysing abdomen colouration. As there was no variation in the number of stripes within males or females, we simply report the number of stripes without further analysis.

#### 2.6.2. Sex differences in metabolomics analysis

We used MetaboScape 2021b (Bruker Daltonics) to process raw data files from UPLC/MS with QC data files as the reference for aligning peaks. This generated a multidimensional peak table (bucket table) that described statistical information such as molecular weight, compound formula, accurate *m*/*z*, retention time and peak area. We combined the peak tables into a single Excel spreadsheet and processed positive ESI data (see electronic supplementary material, table S2). To assign the detected metabolites to a chemical class, we analysed the UPLC/MS data using SIRIUS 4 [44], for fast identification of molecular structure based on isotope patterns and fragmentation trees. We performed exploratory data analysis (hierarchical clustering heatmap) using the heatmap.2() function from the gplots package. The data were first scaled by row (z-score normalization), and then hierarchical clustering was applied to rows (m.z values). This visualization shows the metabolite intensities across different samples and sexes, highlighting patterns and clusters in the metabolomic data and the identification of sex-specific and sample-specific metabolomic signatures. We also calculated fold differences. The mass-to-charge ratios (m.z), intensity values and sex information for each sample were first aggregated by mass-to-charge ratio (m.z) and sex. The mean intensity was then calculated for each group, and the fold change was calculated as the ratio of female to male intensities. We calculated a Log2 fold change to normalize the fold change distribution. The relative intensity was calculated as the higher of the female or male intensity. This enabled us to identify compounds that had at least a tenfold difference between males and females (table 1) and for us to assign a putative chemical class.

**Table 1.** List of 14 compound classes that differed the most in mean intensity between male and female *A. nigriceps* sampled by metabolomic methods, with their subclass, molecular formula, retention time (RT), peptide mass and male and female intensity. Mean intensity refers to the detector response for each molecule. These compounds only consist of those where classification, literature and further information could be found. A full list of found compounds can be found in electronic supplementary material, table S3.

most specific class	subclass	molecular formula	compound identification?	RT	peptide mass	male mean intensity	female mean intensity	notes	references
*N*-acyl-alpha amino acids	amino acids, peptides and analogues	C_24_H_46_N_2_O_3_	oleamido ethylacetamide ethanolate	5.29	411.35764	1650.18	575 779.43	connected with estrogen receptors and endocrinology	https://pubchem.ncbi.nlm.nih.gov/compound/71587381
*N*-acyl-alpha amino acid	amino acids, peptides and analogues	C_24_H_44_N_2_O_3_	termitomycamide D	4.97	409.34201	1175.87	392 624.03	found in fungi originally. Suppresses endoplasmic reticulum stress	https://pubchem.ncbi.nlm.nih.gov/compound/Termitomycamide-D [45]
*N*-acyl amines	fatty amides	C_18_H_35_NO_2_	palmitoleoyl ethanolamide (POEA)	5.87	298.27372	48 787.32	310 816.1	is an endocannabinoid and a *N*-acylethanolamine, but also a compound used in surfactants in pesticides such as glyphosate and found to cause toxicity in insects, mammals and amphibians	https://pubchem.ncbi.nlm.nih.gov/compound/Palmitoleoyl-Ethanolamide [46,47,48]
*N*-acyl-alpha amino acids	amino acids, peptides and analogues	C_20_H_35_NO_3_	N-linoleoyl glycine, glycine linoleamide	6.27	338.2687	3435.97	219 366	has a role as a metabolite. Causes muscle weakness and depressed behaviours in rodents. Induces defences in *Daphnia*. A defence chemical used by plants to avoid herbivory predation, but some insects have adapted to overcome it	https://pubchem.ncbi.nlm.nih.gov/compound/N-linoleoylglycine [49,50,51]
*N*-acyl-alpha amino acids	amino acids, peptides and analogues	C_18_H_35_NO_3_	*N*-palmitoylglycine	6.61	314.26872	678.5	132 774.05	endogenous lipids. It has a role as a marine metabolite and a human metabolite. Located in cellular membrane	https://pubchem.ncbi.nlm.nih.gov/compound/N-Palmitoylglycine [49,52]
phenylalanine and derivatives	amino acids, peptides and analogues	C_27_H_43_NO_3_	imperialine	7.17	430.33098	0	130 129.26	isosteroid plant alkaloid, from *Fritillaria cirrhosa* and other sp. member of the lily family. May cause spasms, vomiting, hypotension and cardiac arrest in humans. But, also used to suppress inflammatory responses in respiratory systems (lungs, coughs etc.)	https://pubchem.ncbi.nlm.nih.gov/compound/Imperialine [53,54]
*N*-acyl amines	fatty amides	C_41_H_79_N_3_O_2_	*N*′,*N*″-dioleylglutamide	6.57	646.62432	252.34	103 604.08	used to create liposomes for administration of pharmaceutical drug delivery	https://pubchem.ncbi.nlm.nih.gov/compound/1021611 [44,55]
*N*-acyl-alpha amino acids	amino acids, peptides and analogues	C_20_H_33_NO_3_	oxeladin	5.91	336.25301	974.48	101 646.05	alkylbenzene. Can cause acute toxicity. Used in the treatment of coughs, but withdrawn from the Canadian, US and UK markets in 1976 due to carcinogenicity. Behavioural: convulsions or effect on seizure threshold; lungs, thorax or respiration; dyspnea in rodents. Effectively suppresses tumours in mice	https://pubchem.ncbi.nlm.nih.gov/compound/Oxeladin [56]
*N*-acyl-alpha amino acids	amino acids, peptides and analogues	C_20_H_37_NO_3_	*N*-oleoyl glycine, rociverine	6.69	340.28421	0	81 339.63	believed to be an intermediate in oleamide biosynthesis. It has a role as a metabolite. Signalling molecule	https://pubchem.ncbi.nlm.nih.gov/compound/N-oleoylglycine [57]
phenylalanine and derivatives	amino acids, peptides and analogues	C_27_H_41_NO_3_	sipeimone	6.82	428.31554	426.66	73 106.44	possibly associated with plant alkaloids	https://pubchem.ncbi.nlm.nih.gov/compound/16395931 [58]
*N*-acyl-alpha amino acids	amino acids, peptides and analogues	C_18_H_35_NO_4_	undecanoylcarnitine	6.07	312.25319	743.18	72 514.25	found in the extracellular membrane. Involved in beta-oxidation	https://pubchem.ncbi.nlm.nih.gov/compound/Undecanoylcarnitine [59]
*N*-acyl amines	fatty amides	C_38_H_72_N_2_O_2_	ethylene bis(oleamide) *N*,*N*′-ethylenebisoleamide	6.7	589.56664	39 087.6	80.06	used in plastic manufacturing—no other information found	https://pubchem.ncbi.nlm.nih.gov/compound/Ethylene-dioleamide
*N*-acyl amines	fatty amides	C_40_H_74_N_2_O_2_	2-(2-heptadec-8-enyl-4,5-dihydroimidazol-1-yl) ethyl octadec-9-enoate	7	615.58223	49 116.86	873.61	an oleic acid. Corrosive and acute toxicity—harmful if swallowed and very toxic to aquatic life. No other classification or literature found	https://pubchem.ncbi.nlm.nih.gov/compound/3019087
alpha amino acids and derivatives	amino acids, peptides, and analogues	C_6_H_7_B_2_NO_3_	SCHEMBL12254162 (4-iminoboranyloxyphenyl) boronic acid	2.95	162.0761	170 049.79	8873.85	contains a pyridine compound, which are known for their ‘unpleasant fish-like’ smell	https://pubchem.ncbi.nlm.nih.gov/compound/59330200 https://pubchem.ncbi.nlm.nih.gov/compound/Pyridine

#### 2.6.3. Sex differences in ecotoxicology

We used two approaches to investigate *Daphnia* survival in the toxicity assays. First, we used a mixed-effects Cox proportional hazards model to compare toxicity (i.e. *Daphnia* survival curves) between male and female moths, using the coxme [60] package. Time before death was explained by moths’ sex and weight, and moth ID was included in the model as a random effect. The proportional hazards assumptions were checked based on the Schoenfeld residuals using the cox.zph function in the survival package [61]. When including all observations, the assumptions were not met for ‘moth weight’ covariate, but this was driven by two outlier moths that were considerably heavier than others, and the assumptions were supported when these two individuals were excluded. However, excluding these moths did not change the main results, and below, we report the results from the model that includes all observations. Second, we used the number of dead *Daphnia* after two hours as an indicator of toxicity. This time point was chosen as it showed the most variation in toxicity among moth individuals (2 hours: average mortality of *Daphnia* 54%). In the prior time point (1 hour), the toxic effects were relatively small (1 hour: average mortality 22%), and after it (3 hours), most *Daphnia* had died (3 hours: average mortality 75%), and we might have therefore missed individual variation in toxicity. The toxicity effects after 2 hours were analysed using a generalized linear model with a Poisson error distribution. The model included the number of dead *Daphnia* after 2 hours as a response variable, and this was explained by a moth’s sex and mass. To incorporate the water and methanol controls, we ran a separate model that included treatment (moth, water control and methanol control) as the only explanatory variable. We also used the 2-hour time point to estimate the repeatability of the assays. This was calculated from a generalized mixed-effects model that included moth ID as a random effect, using the rptR package [62]. The analyses were conducted with R.4.4.3 [63].

## 3. Results

### 3.1. Sex differences in morphology and warning signals

There was a significant interaction between sex and location on wing length (sex × population: estimate = −0.815 ± 0.277, *t* = −2.942, *p* = 0.004). In Wyoming, males had longer wings than females (estimate = 0.845 ± 0.222, *t* = 3.800, *p* < 0.001), but this difference did not occur in the moths from Macquarie Park (estimate = 0.030 ± 0.165, *t* = 0.180, *p* = 0.86). Both females (estimate = −1.690 ± 0.240, *t* = −7.029, *p* < 0.001) and males (estimate = −0.875 ± 0.138, *t* = −6.345, *p* < 0.001) had shorter wings in Wyoming compared to Macquarie Park.

**Figure 2.**
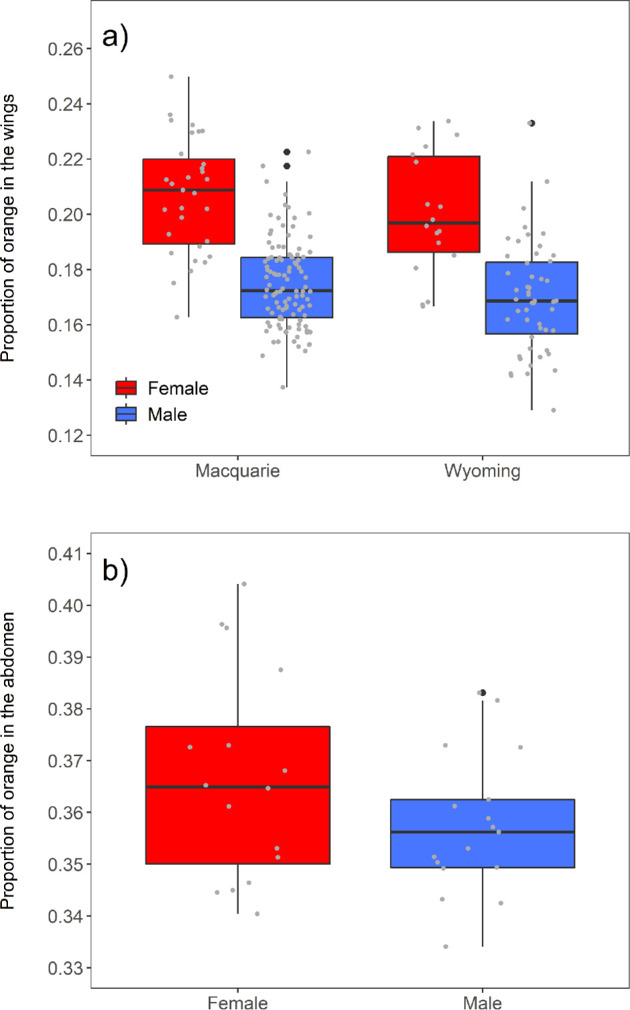
Sex differences in the warning colouration of *A. nigriceps.* (*a*) The proportion of orange in the wings (*pSpot)*. The moths were collected from two different locations (Macquarie Park: *n* = 139, Wyoming: *n* = 69). (*b*) The proportion of orange in the abdomen (*pStripe*). The moths were collected from the same two locations (Macquarie Park: *n* = 21, Wyoming: *n* = 12) but because of a smaller sample size, these are not plotted separately. The box plots illustrate the median and 25th and 75th percentiles, the whiskers indicate the values within 1.5 times the interquartile range, the grey circles represent individual variation and the black circles are outliers.

The proportion of orange in the wings (*pSpot*) varied from 0.13 to 0.25 among individuals. Females had a higher proportion of orange in the wings than males (estimate = 0.031 ± 0.003, *t* = 10.572, *p* < 0.001; figure 2a). This difference was similar in both locations (sex × location: estimate = 0.001 ± 0.006, *t* = 0.215, *p* = 0.83), and the interaction term was removed from the final model. We found a significant difference between the locations, with moths from Macquarie Park having a higher proportion of orange in the wings than those from Wyoming (estimate = 0.006 ± 0.003, *t* = 2.069, *p* = 0.04; figure 2a). All the females sampled (*n* = 48) had five abdominal stripes and all the males sampled (*n* = 159) had six abdominal stripes. The proportion of orange in the abdomen (*pStripe*) varied from 0.33 to 0.40 among individuals, and this did not differ between the sexes (estimate = 0.009 ± 0.007, *t* = 1.343, *p* = 0.19; figure 2b) or the locations (estimate = 0.0002 ± 0.007, *t* = 0.030, *p* = 0.98).

### 3.2. Sex differences in chemical defences

**Figure 3.**
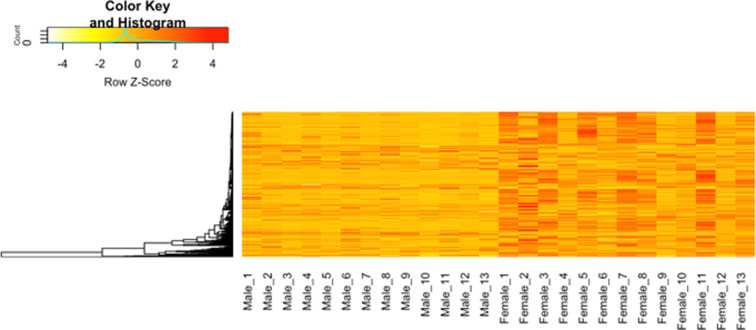
Hierarchical clustering heatmap representing the metabolite intensities of male and female replicates. The rows correspond to the *m*/*z* (mass-to-charge ratio) values of the metabolites, and the columns represent individual replicates, with 13 male and 13 female samples. The intensity values have been *z*-score normalized across each row (metabolite), with hierarchical clustering applied to the rows. Warmer colours (red and orange) indicate higher relative intensities, while cooler colours (yellow and white) represent lower intensities.

**Figure 4.**
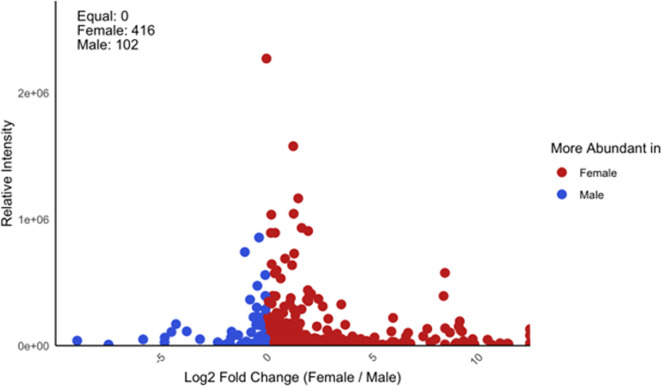
Log2 fold change in relative intensity of metabolites across male and female samples of *A. nigriceps*. Each point represents a metabolite characterized by its *m*/*z* (mass-to-charge ratio). Red dots indicate metabolites more abundant in females (*n* = 416) and blue dots indicate those more abundant in males (*n* = 102).

Our targeted metabolomics method did not detect PA. The untargeted metabolomics approach detected 474 compounds (electronic supplementary material, table S3). Eleven compounds were exclusively expressed in females, and 107 compounds were at least 10 times greater in females than males (figures 3 and 4), while only eight compounds in males were 10 times greater than in females (electronic supplementary material, table S3). No compound was exclusively expressed in males. Of the 107 compounds that differed at least 10-fold, we were able to putatively assign identities to 14 compounds using the PubChem database (table 1). While many chemicals that differed between males and females can be associated with reproductive physiology (e.g. *N*-acyl-alpha amino acids; electronic supplementary material, table S3), cellular metabolism (e.g. *N*-acyl-alpha amino acids; electronic supplementary material, table S3), neurotransmitters (e.g. glycine) and signalling molecules such as nitric oxide (NO), notably there are steroidal alkaloids associated with plant chemical defences (table 1).

### 3.3. Ecotoxicology

**Figure 5.**
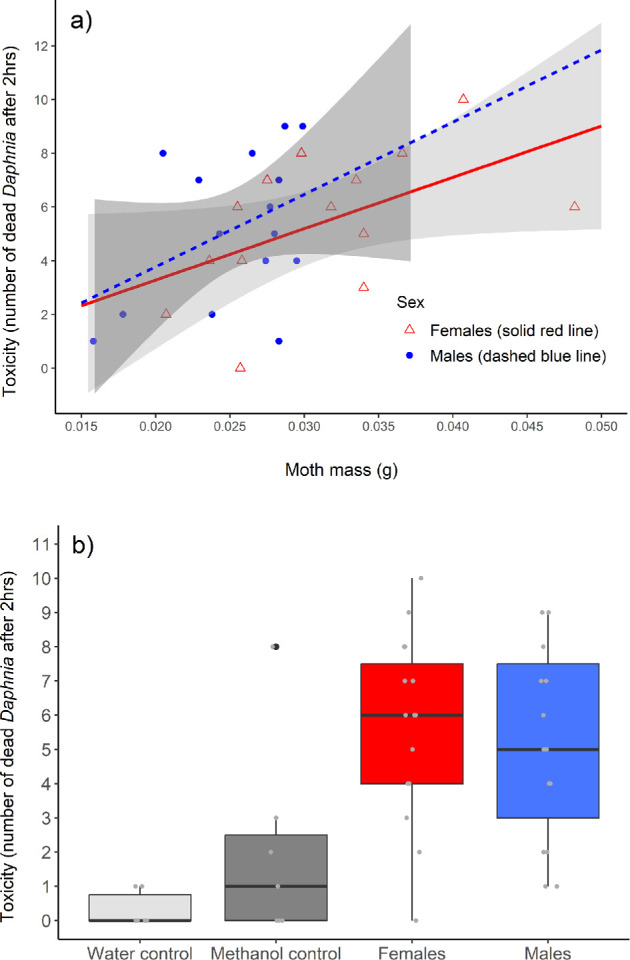
Results of ecotoxicology assays. (*a*) Toxicity (number of dead *Daphnia* after 2 hours) did not differ between female (*n* = 15, red triangles and solid line) and male moths (*n* = 15, blue circles and dashed line), but depended on the mass of the moth. Shaded areas around the lines indicate 95% CIs for predictions from linear models. One individual that weighed 0.066 g was excluded from the figure for illustrative purposes (but it was included in the analysis). (*b*) Both male and female moths were more toxic compared to the water (*n* = 6) and methanol controls (*n* = 7). The box plots represent the median and 25th and 75th percentiles, the whiskers indicate the values within 1.5 times the interquartile range and the black circle shows an outlier in the methanol control group.

We found no significant difference in toxicity between female and male moths based on *Daphnia* survival (CoxPH: effect = 0.114 ± 0.178, *Z* = 0.64, *p* = 0.52; figure 5). There was a significant effect of mass, with heavier moths being more toxic than lighter moths (CoxPH: effect = 43.442 ± 9.074, *Z* = 4.79, *p* < 0.001; figure 5a). The analysis of *Daphnia* mortality after 2 hours provided similar results, with no significant difference between the sexes (GLM: effect = 0.135 ± 0.180, *Z* = 0.752, *p* = 0.45) and a significant effect of mass (GLM: effect = 22.834 ± 7.752, *Z* = 2.945, *p* = 0.003). The repeatability of the assays was high (*R* = 0.93, 95% CI: 0.86−0.97). The methanol control was more toxic than the water control (GLM: effect = 1.792 ± 0.756, *Z* = −2.370, *p* = 0.018), and both controls were less toxic compared to the moths (water: effect = −2.791 ± 0.711, *Z* = −3.923, *p* < 0.001; methanol: effect = −0.999 ± 0.279, *Z* = −3.589, *p* < 0.001; figure 5b).

## 4. Discussion

Our study investigated morphological and chemical differences of red-necked wasp moths (*A. nigriceps*) from two locations in NSW, Australia. We found sexual dimorphism in the warning signals, with females having significantly larger proportions of orange in their wings than males, and this held true across both locations. This is in line with our previous research which also found variation in the proportion of orange in the warning signal between males and females [21]. The number of orange abdominal stripes also differed between the sexes, with females always having one fewer stripe than males. This is likely due to anatomical modifications to the tip of the female abdomen that are necessary to accommodate female genitalia [64]. While we ruled out the presence of PA in adult moths [31–33], we found that females contained higher amounts of potential secondary metabolites than males. Despite the differences between the sexes in the metabolomics analysis, we did not find toxicity differences in the ecotoxicology assays using *Daphnia*.

Across the two locations where we collected adult moths, we found a significant difference in the proportion of orange between Macquarie Park and Wyoming, although the difference is very small and of unknown biological relevance. We selected moths collected at two populations that were ~60 km apart to match our previous work where we found no evidence for phenotypic plasticity of *A. nigriceps* wing pattern elements in response to seasonal or rearing temperature [21]. So, it is unlikely that any differences in temperature between these two localities explain the difference in wing colour patterns. Moths from Macquarie Park were also larger than those from Wyoming. It is possible that the different levels of land change at these two sites are driving the observed differences in the proportion of orange in the wing. For example, Merckx *et al*. [65] have shown that increased levels of urbanization are associated with larger individuals within species of macro-moths. Given the changes in urbanization across Australia and NSW, it would be interesting to determine whether urbanization can be related to variation in warning signals and wing size in this and other aposematic Lepidoptera.

We also found that in moths collected in Wyoming, males had larger wing lengths than females, while those from Macquarie had comparable wing lengths between the sexes. In Lepidoptera, wing size and aspect ratio are important components of flight performance and are positively related to acceleration [66] and dispersal [67], but reduce manoeuvrability [68]. Mobility and dispersal are known to be under strong selection in Lepidoptera [69]. Whether the differences in wing size and colour patterns between male and female *A. nigriceps* are linked to variations in flight and related behaviours remains to be tested. Though we have observed that females tend to be less agile in flight than males and that they are more likely to hide under or behind vegetation compared to males (G.E.B., personal obs.).

We have previously shown that warning signal variation in *A. nigriceps* can be explained by differential selection pressures on individual colour pattern elements by experienced avian predators [22]. When these signals are moving, and dynamic, spots on the wings might appear blurred to a predator due to motion during flight and abdomen stripes might be more stable [70]. We propose that the proportion of orange on the wings and the number of abdominal stripes may serve different functions during flight and rest, potentially influencing other morphological aspects of the wings if these traits interact during signalling.

In addition to sex difference in the expression of orange, we also found a considerable difference in the metabolites, which may include various toxins present in males and females. One of the potential toxins we specifically searched for were PA that have been reported to accumulate in arctiid moths [31–33,71], and we explored the possibility that this class of compounds also plays a role in the chemical defence of *A. nigriceps*. While we found substantial differences in both the types and quantities of compounds present in males and females, we did not find PA. This may indicate that *Amata* moths do not feed on plants that contain PA or that moths from the localities we sampled lack them. Information on the host plant(s) used by *A. nigriceps* will be important to further investigate the nature and development of their chemical defences. We putatively identified two classes of compounds that differ between males and females: *N*-acyl-alpha amino acids and *N*-acyl amines. These chemicals are conjugates of fatty acids (with various degrees of unsaturation), either with amino acids or with various amines. Generally, they function as signalling molecules or neurotransmitters [72,73]. They could also be involved in cuticle formation. The compounds of note are *N*-linoleoyl glycine and imperaline compounds (table 1) that have been linked to plant defence toxins [50] and plant alkaloids [53], respectively, as well as any of the biogenic amines we detected [74]. These were either found exclusively in females or present in significantly higher amounts in females (table 1). Our analysis is, however, based on molecular formulas, retention times and masses, which cannot provide definitive identifications. Additional spectroscopic data would be needed for more confident assignments.

Despite the potential differences in metabolite profiles of males and females, we found no significant difference in ecotoxicology assays between the sexes. While *Daphnia* are not natural predators of *Amata* moths, they are a commonly used model species in ecotoxicology assays (see [34,43]). More ecologically relevant results of this moth’s toxicology would require tests of unpalatability/toxicity against natural avian (or other) predators. While there were no toxicity differences between the sexes, we found high variation among individuals, which would be interesting to relate to the proportion of orange in the wing in future studies to determine potential trade-offs between signalling and toxicity [11,13–17].

We were not able to control for age or mating status in our wild-caught animals and thus limit this source of variation in the different quantities of metabolites and toxicity we found within each sex. One source of individual differences is likely the sequestration of toxins during the juvenile growth period, as adults mostly feed on nectar (G.E.B., personal observation). Other sources include toxin synthesis [75] or transfer of toxin by the male with the spermatophore (e.g. [71]).

## 5. Synthesis and speculation

Why then does the warning signal pattern of *A. nigriceps* vary between sexes if there is no significant difference in toxicity, but production of a higher proportion of orange is associated with lower predation costs [22]? One possibility is that there may be sex-specific predation pressure that is modulated by a different mode of defence that we have not measured. *Amata* moths secrete a neck fluid that has a potentially defensive odour [20]. During extraction, we noted a strong pyrazine-like odour of the moths (L.C. and H.M.R., personal observations), and this warrants further investigation. In other aposematic tiger moths (e.g. *Arctia plantaginis*), this neck fluid contains pyrazines that are effective at deterring bird predators [76]. The proportion of orange in the wings, however, has a high heritability (h2 = 0.7 [21]) and traits associated with sex-specific differential selection tend to have lower heritability [77–81]. A second possibility is that even though larger orange warning signals are more effective against predators [22], this benefit in *A. nigriceps* may trade off against thermoregulatory benefits arising from larger black pattern elements [7] or immunity against pathogens [82]. Darker colouration can also protect individuals from UV radiation [83]. A third and related possibility is that variation in the size of pattern elements is maintained by resource availability or condition dependence (e.g. [13,16,84]). To test this, we would need more robust data on the pigments, secondary defences and food plants of the moths to understand the limitations that might be imposed on the different pattern elements. A fourth possibility is that there are opposing selection pressures on signal size. There is increasing focus on and evidence of the close interplay between natural and sexual selection on warning colour signals [23,28]. If aposematic signals contribute to both predator deterrence and mate attraction, these interactive effects could drive sexual dimorphism in warning signals or maintain signal variation within one sex [28]. Future research should explore the possibility of assortative mating in *A. nigriceps.* Finally, despite our unambiguous results, we cannot exclude that certain predators can distinguish the taste or toxicity between the two sexes. We only have some anecdotal account of a great tit trying a male *Amata* moth and showing a very strong disgust reaction (J.M., personal observation). Testing potential predators and their responses is an obvious next step for understanding this aposematic system.

Before these more targeted empirical tests are possible, more information about the chemical composition of the pigments and secondary defences used by aposematic species is needed. As shown here, visual and chemical analysis of aposematic traits can propose new lines of research into the complex interactions involved in expression of warning signals.

## Data Availability

All data (with READMEs) are accessible online [85]. Supplementary material is available online [86].

## Authors' Notes

We strongly support Equity, Diversity and Inclusion in Science [87]. Researchers included in this study are in different stages of their careers, from PhD student through to early and senior researchers. Our gender balance is biased towards women; at least three authors identify as part of the LGBTQI+ community or have a disability; more than one author is a first-generation university graduate and remains the only member of their family to have entered higher education. Our authors come from different countries such as Australia, Finland, Austria, Italy, Ecuador and the United Kingdom.
